# The relationship between wasting and stunting: a retrospective cohort analysis of longitudinal data in Gambian children from 1976 to 2016

**DOI:** 10.1093/ajcn/nqy326

**Published:** 2019-02-07

**Authors:** Simon M Schoenbuchner, Carmel Dolan, Martha Mwangome, Andrew Hall, Stephanie A Richard, Jonathan C Wells, Tanya Khara, Bakary Sonko, Andrew M Prentice, Sophie E Moore

**Affiliations:** 1 Medical Research Council (MRC) Elsie Widdowson Laboratory, Cambridge, UK; 2 Emergency Nutrition Network, Oxford, UK; 3 Kenya Medical Research Institute (KEMRI)‐Wellcome Trust Research Programme, KEMRI Centre for Geographic Medicine Research‐Coast, Kilifi, Kenya; 4 Independent Consultant, UK; 5 Fogarty International Center, National Institutes of Health, Bethesda, MD; 6 Childhood Nutrition Research Centre, University College London Great Ormond Street Institute of Child Health, London, UK; 7 MRC Unit The Gambia at the London School of Hygiene and Tropical Medicine, London, UK; 8 Department of Women and Children's Health, King's College London, London, UK

**Keywords:** wasting, stunting, concurrently wasted and stunted, growth-monitoring data, seasonality, boys, girls

## Abstract

**Background:**

The etiologic relationship between wasting and stunting is poorly understood, largely because of a lack of high-quality longitudinal data from children at risk of undernutrition.

**Objectives:**

The aim of this study was to describe the interrelationships between wasting and stunting in children aged <2 y.

**Methods:**

This study involved a retrospective cohort analysis, based on growth-monitoring records spanning 4 decades from clinics in rural Gambia. Anthropometric data collected at scheduled infant welfare clinics were converted to *z* scores, comprising 64,342 observations on 5160 subjects (median: 12 observations per individual). Children were defined as “wasted” if they had a weight-for-length *z* score <–2 against the WHO reference and “stunted” if they had a length-for-age *z* score <–2.

**Results:**

Levels of wasting and stunting were high in this population, peaking at approximately (girls–boys) 12–18% at 10–12 months (wasted) and 37–39% at 24 mo of age (stunted). Infants born at the start of the annual wet season (July–October) showed early growth faltering in weight-for-length *z* score, putting them at increased risk of subsequent stunting. Using time-lagged observations, being wasted was predictive of stunting (OR: 3.2; 95% CI: 2.7, 3.9), even after accounting for current stunting. Boys were more likely to be wasted, stunted, and concurrently wasted and stunted than girls, as well as being more susceptible to seasonally driven growth deficits.

**Conclusions:**

We provide evidence that stunting is in part a biological response to previous episodes of being wasted. This finding suggests that stunting may represent a deleterious form of adaptation to more overt undernutrition (wasting). This is important from a policy perspective as it suggests we are failing to recognize the importance of wasting simply because it tends to be more acute and treatable. These data suggest that stunted children are not just short children but are children who earlier were more seriously malnourished and who are survivors of a composite process.

## Introduction

Undernutrition during the early years of life has a harmful and irreversible impact on child development and is a major influence on the risk of dying throughout life (1). Each year, ∼800,000 deaths are in part attributable to wasting, 60% of them attributable to being severely wasted. In addition, >1 million deaths are attributable to stunting, but this association remains poorly understood. In the 1980s, an economist suggested that short children of adequate weight should be considered “small but healthy,” and did not merit nutritional intervention (2). This view is now rejected, given evidence linking short stature with poor cognitive development in childhood, and with adult noncommunicable disease risk, but the elevated child mortality associated with stunting remains an enigma (3, 4).

Although progress is being made in decreasing undernutrition in low- and middle-income countries, stunting and wasting during childhood continue to burden people in the poorest regions in the developing world. Both these outcomes of undernutrition occur together in children in many contexts and may co-occur in the same child (referred to as “concurrence” throughout this paper). However, wasting or stunting is often considered separately as outcomes of undernutrition, notably with respect to how they are managed (clinically and programmatically) (5, 6) and in how they are researched. Despite recognized distinctions in their etiology, it is this historical separation that underpins the rationale for the analysis reported in this paper. Furthering our understanding of the relationship between these 2 outcomes of undernutrition may help us to identify opportunities to optimize our efforts, both in treatment and prevention programming, to better affect both outcomes through aligning particular aspects of programming.

Analyses of the processes of stunting and wasting suggest that they share many common risk factors (7) including poor maternal health and nutrition, inadequate infant and young child feeding practices, poor diet, and frequent bouts of infection. Becoming stunted is largely considered to be irreversible after the age of 2 y, although periods of “catch-up” growth, e.g., in adolescence, have been described (8). Research efforts have largely focused on the development of interventions to prevent children from becoming stunted, although, to date, both nutrition-specific and nutrition-sensitive interventions are yielding disappointing impacts. For example, combinations of interventions to improve complementary foods with and without maternal education show an average efficacy of <0.3 *z* scores of height-for-age, which amounts to ∼1/6 of the usual deficit compared with WHO Growth Standards (9). Further, a recent meta-analysis of previous interventions for water, sanitation, and hygiene showed only a marginally significant effect on height-for-age (0.08 *z* scores; 95% CI: 0.00–0.16) and no beneficial effect on weight-for-height or weight-for-age (10). Unlike stunting, wasting appears to be short term in nature and reversible if the child has an adequate diet, is protected from infectious diseases, and has access to medical treatment (11). However, although wasting is widely viewed as an acute problem, a child may experience repeated episodes of wasting in their early years that carries a greater risk of dying and may lead to stunting in the long term (11).

The rationale behind the conceptual separation of stunting and wasting in terms of etiology and programs has been questioned in a number of recent reviews and publications (6, 12). These publications highlight outstanding gaps in our understanding of the interrelationship between these 2 forms of undernutrition as a result of insufficient examination of data from longitudinal cohorts in particular. Here, we contribute to filling that gap by describing the interrelationships between wasting and stunting in rural Gambian children <2 y of age, based on data collected over 4 decades of scheduled longitudinal growth monitoring in >5000 children.

## Methods

### Study population

We conducted a retrospective cohort study that used high-quality, clinic-based routine growth-monitoring data from community children in the rural villages of Keneba, Manduar, and Kantong Kunda, collected by the UK Medical Research Council (MRC) Keneba clinic (13). These villages are in the rural West Kiang region of the Gambia, where the population relies primarily on subsistence agriculture. Food availability fluctuates widely across the year and the wet season, lasting from July to October, which is a “lean” period because stored staple foods from the previous year's harvest are nearly depleted. At the same time, adults have an increased workload to prepare for the current year's harvest. The dry/harvest season is a time of relative plenty. More broadly, seasonal factors in this environment contribute to many aspects of variability in nutrition, health, and behavior in both adults and children (14).

The data used in the current analysis were collected at scheduled infant welfare clinics at the MRC Keneba clinic between May 1976 and September 2016. This population has been described in detail elsewhere (13, 15, 16), and this longitudinal data have previously been used to demonstrate a significant reduction in rates of both stunting and underweight (but not wasting) over the past 4 decades (15). Briefly, 3 rural villages in this region (Keneba, Kantong Kunda, and Manduar) have been part of a demographic surveillance project for the past 40 y. As part of this program, the villagers have benefited from free health care provided by the MRC and from increasing levels of support and interventions, including a center offering nutritional rehabilitation for children with severe acute malnutrition (15). Clinic-based growth monitoring of young children was done monthly in the 1970s, but from 1983 onwards, anthropometric measurements were made at birth, 6 wk, 3 mo, and then every 3 mo thereafter. These measurements were conducted in infant welfare clinics, by trained health care workers who used standardized protocols and regularly validated equipment. Carers were notified in advance of their appointment, and transport was provided by the MRC to bring mothers and their infants to the clinic. These measurements therefore represent an unselected record of growth because children are measured regardless of their health status at the time of their clinic call. In addition to anthropometric measurements, these visits also included health assessments, advice on feeding practices and diet, and the administration of vaccines (according to the Gambian Government schedule at that time). Ethical approval for the longitudinal demographic surveillance and clinical data collection of the 3 villages was granted by the Joint Gambian Government/MRC Unit The Gambia Ethics Committee (13).

In the analysis presented here, we tested 3 broad research questions: *1*) Is wasting a risk factor for stunting, and vice versa? *2*) Does the season of birth influence future wasting and stunting? *3*) Are there gender differences in growth deficits in the Gambia?

### Statistical methods

**FIGURE 1:**
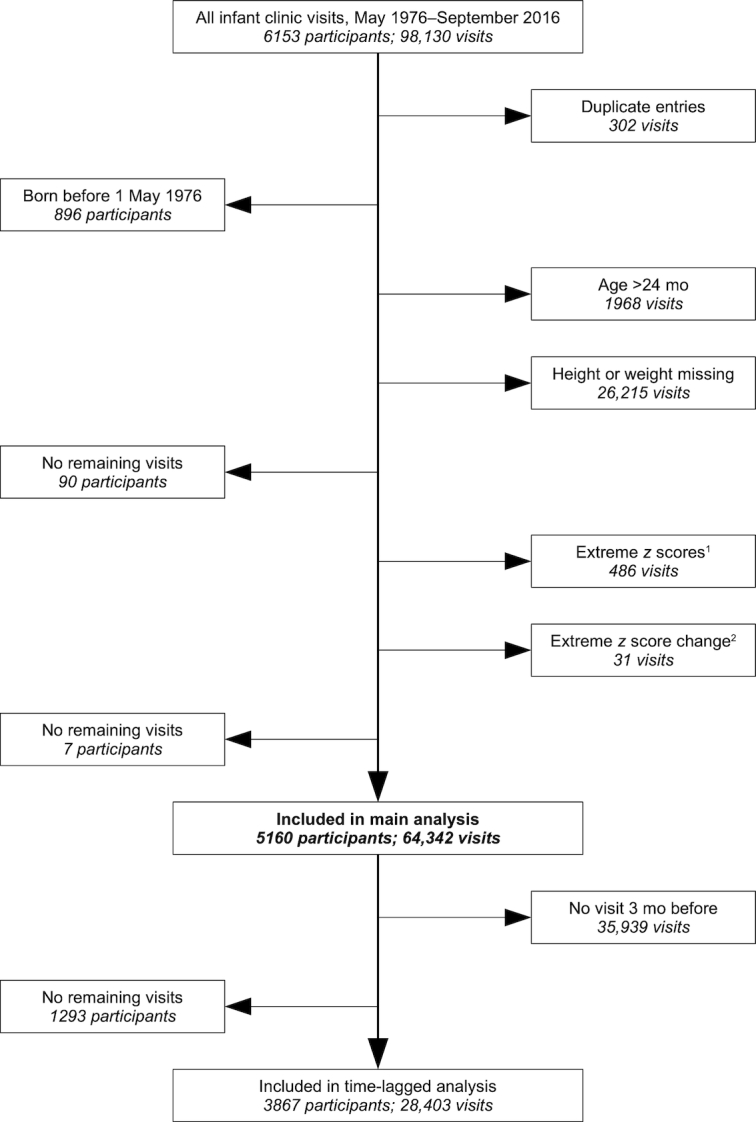
Flow diagram of sample selection from data collected at scheduled infant welfare visits at the MRC Keneba clinic. A distinction is made between excluded participants (left) and excluded visits (right); when the criteria on the right led to exclusion of all of a participant's visits, that participant was excluded because he/she had no remaining visits to include. MRC, Medical Research Center. ^1^Extreme *z* scores excluded as per WHO criteria (see Statistical methods). ^2^Extreme *z* score change of >3 SDs between consecutive visits, outlying observation excluded.

Statistical analysis was carried out in R version 3.3.2 (17) primarily with the use of the following packages: dplyr version 0.5.0 for data manipulation (18), lme4 version 1.1.12 for multilevel modeling (19), and ggplot2 version 2.2.1 for plotting (20). Anthropometric indices were calculated according to the WHO (2006) growth standards (21). Wasted was defined as a weight-for-length *z* score (WLZ) <−2, stunted as a length-for-age *z* score (LAZ) <−2, and a small mid-upper arm circumference (MUAC) as <12.5 cm. After excluding implausible *z* scores (LAZ >6 or <−6 or WLZ >5 or <–5), the data comprised 64,342 observations on 5160 subjects (**Figure 1**; median 12 observations per individual). The statistical methods used are described separately for each research question below.

### Cross-sectional prevalence rates

To describe age-related differences in the prevalence of stunting, wasting, or concurrence (i.e., WLZ <−2 and LAZ <−2), the longitudinal data were split into 1-mo age groups (by completed month of age from 0 to 23 mo). If an individual had >1 measurement in 1 mo, they were classified as wasted, stunted, or both based on their mean *z* score within that month.

### Repeated episodes of wasting in consecutive wet seasons

To estimate whether infants who were wasted in their first wet season tended also to be wasted in the subsequent wet season, a subset (*n* = 2902) was selected according to the following criteria: ≥1 observation during the first wet season of life (season of birth if born during the wet season), the second wet season, and the dry season in between. Within a season, each participant's median WLZ was used to classify them as wasted or not wasted. The relationship between wasting during those 3 seasons was estimated through the use of logistic regression, with being wasted in the second wet season as the outcome, and being wasted in the first wet season and the intermediate dry season as binary predictors. Wasting in the dry season was included as a predictor to distinguish subjects who were wasted continuously (year-round) from those who experienced seasonal episodes of wasting.

### Early growth trajectories and subsequent risks of stunting

To investigate the impact of early patterns of growth on subsequent risk of stunting, growth curves were developed and fitted separately for subjects who were stunted between the ages of 20 and 24 mo and those who were not. Age- and season-related *z* score trajectories were modeled as smooth curves: natural cubic splines for the age-related change and second-order Fourier terms for the seasonal changes. An interaction was included to allow for age-related differences in the magnitude of the seasonal variation. Random intercepts and slopes were included to allow for individual-level variation around the mean growth curves (representing individual differences in size and velocity). Trajectories were then plotted for comparison, but no formal statistical tests were carried out to analyze differences between the stunted/nonstunted groups.

### Individual weight-for-length trajectories as predictors of subsequent risk of stunting

To determine whether features of the individual WLZ trajectories were predictive of becoming stunted, we then extracted individual-level random effects from trajectories modeled by multilevel regression within each sex, but without prior separation of the stunted/nonstunted groups. In addition to random effects for the intercept and slope, random effects were included for the first-order Fourier terms, allowing the amplitude of seasonal variation to vary between individuals. Thus, individual intercepts, slopes, and seasonal terms could be extracted from the models. Each subject's seasonal random effects were summarized through the use of the coefficient of cyclic variation (CCV) (22). This describes the magnitude of seasonal variation in each growth outcome, above or below the seasonal variation of the mean trajectory. Logistic regression was used to predict the risk of being stunted between the ages of 20 and 24 mo as a binary outcome, based on previous episodes of being stunted or wasted and the extracted random effects as predictors.

### Longitudinal prediction of stunting through the use of time-lagged wasting

Finally, instead of predicting stunting at 20–24 mo of age as an individual-level, cross-sectional outcome, the risk of becoming stunted was also predicted longitudinally based on the use of time-lagged observations of prior episodes of stunting or wasting. This provides an age-dependent prediction. A time lag of 3 mo was chosen based on the observation made as part of the previous analysis that seasonal variation in height occurs ∼3 mo later than seasonal variation in weight.

Each observation was matched 1-to-1 with a prior observation on the same individual, chosen to be as close as possible to 3 mo earlier. If that individual was not measured between 2.5 and 3.5 mo before, then the current observation was dropped. This left a total of 28,403 observations on 3867 subjects (Figure 1; median 7 observations per individual). Multilevel logistic regression was used to describe the relations between being currently stunted (binary outcome) and the time-lagged predictors: age, stunted, and wasted. Stunting was included as a time-lagged predictor to estimate whether being wasted has any predictive power beyond that of knowing whether the individual was already stunted 3 mo previously. Age and sex were included in each model to account for differences in the risk of stunting. The inclusion of 2-way interaction terms did not improve the model fit, and therefore these are not reported.

## Results

### Cross-sectional prevalence rates

**FIGURE 2:**
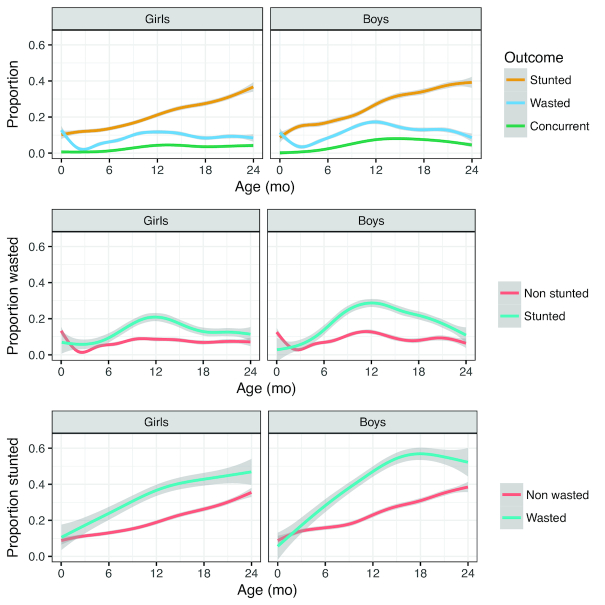
Stunting, wasting, and concurrence by age; cross-sectional estimates expressed as proportions, smoothed by local regression with 95% CIs, *n* = 5160 participants, 64,342 visits. Narrow CIs are obscured by the mean line. The top panel shows overall proportions as in Table 1. The middle and lower panels group the observations by current stunting and wasting, respectively, illustrating that wasting is more common among those who are also stunted than among those who are not, and vice versa.

The cross-sectional prevalence of wasting, stunting, or concurrence by sex in each 1-mo age group is shown in **Table 1**. The proportions are also plotted in **Figure 2**, smoothed across the age range by local regression to illustrate the age-related differences more clearly. The prevalence of stunted children increased with age, up to 39% in both sexes at age 24 mo. Wasting showed an early decline in the first 3 mo (reflecting a period of positive weight gain in the months immediately postpartum), followed by a peak at ∼1 y of age (18% in boys, 12% in girls). The prevalence of children with concurrence peaked at 9% in boys and 5% in girls, also around the age of 1 y.

**TABLE 1:** Participants stunted, wasted, and concurrently wasted and stunted, percentage by age^1^

	Girls				Boys			
Age, mo	*n*	Stunted	Wasted	Concurrent	*n*	Stunted	Wasted	Concurrent
0	1024	8.4	9.3	0.7	1121	8.5	8.7	0.4
1	1344	12.3	3.3	0.9	1347	12.8	4.0	0.8
2	1261	11.0	2.6	0.6	1411	14.8	3.8	0.4
3	1382	11.7	3.2	0.5	1405	14.4	4.6	0.9
4	927	12.1	4.3	0.9	1034	15.7	6.2	1.4
5	1325	12.8	5.6	0.9	1385	16.1	6.2	1.8
6	1047	13.7	6.5	1.7	1161	17.7	9.0	2.7
7	868	15.4	6.8	1.5	925	19.9	11.1	3.8
8	1358	16.4	9.9	2.8	1456	18.5	12.6	4.7
9	845	16.0	11.1	2.6	903	20.9	15.1	6.0
10	1307	18.8	9.8	4.1	1403	23.0	15.2	5.8
11	976	20.1	12.2	4.6	1078	26.0	16.4	7.4
12	963	22.4	11.3	4.2	1074	28.1	18.5	8.8
13	612	22.5	10.5	5.2	698	30.2	17.0	8.9
14	904	26.4	11.3	4.8	930	31.5	13.4	7.4
15	918	24.9	9.6	3.7	1040	33.4	13.4	8.0
16	624	25.5	9.0	3.8	684	31.0	13.9	7.2
17	856	27.3	8.3	2.8	903	32.1	12.2	7.1
18	1008	28.5	7.6	3.5	1039	36.7	13.0	8.3
19	545	26.2	11.0	4.6	572	35.1	13.6	7.0
20	860	30.3	8.0	3.4	893	36.8	11.3	6.7
21	775	33.4	9.9	4.5	866	38.7	13.2	6.5
22	521	31.9	8.6	4.4	519	37.8	11.4	5.6
23	727	37.3	8.8	4.0	754	39.1	7.8	4.1

1 Data from *n* = 5160 participants, with repeated measures grouped by completed months of age. Where a subject contributed multiple measurements in the same age group, their mean *z* score was used to classify them as stunted, wasted, or both stunted and wasted, and *n* was adjusted accordingly. Wasting was defined as weight-for-length <−2 SD, stunting as length-for-age <−2 SD.

The relationship between wasting and stunting is illustrated in the middle and lower panels of Figure 2, based on the same cross-sectional data shown in Table 1. In the middle panel, the proportion of wasted children at each month of age was plotted for children who were either stunted or not by age 20–24 mo. Generally, compared with nonstunted children, a larger proportion of children who were stunted at age 20–24 mo had experienced prior episodes of wasting. The lower panel shows the same relationship in reverse: the proportion of stunted children by age and sex separated by whether they were wasted or not. A greater proportion of wasted children than nonwasted children was stunted after the age of ∼3 mo, and this proportion increased more rapidly among boys than girls. The proportion of wasted children who were also stunted starts to level off from 12 mo in girls and to decline at ∼16 mo in boys.

### Repeated wasting in consecutive wet seasons

Infants who were wasted in the first wet season of their life were more likely to be wasted in their second wet season, even after controlling for whether they were wasted during the intervening dry season (OR: 3.2; 95% CI: 2.3, 4.4) (**Table 2**). This means that infants who were wasted in their first wet season were more likely to be wasted again in their second season, even if they had temporarily recovered in between. As might be expected, a higher correlation was observed between wasting in the dry season and wasting in the second wet season [OR: 12 (95% CI: 8, 17)], indicating that a child who was stunted in the dry season would still be stunted in the wet season immediately afterwards.

**TABLE 2:** Odds ratios of wasting in the second wet season of life, based on wasting in the first wet season and in the intervening dry season^1^

	Wasted in second wet season
Baseline odds	0.12 (0.10, 0.13)
Wasted in first wet season	3.2 (2.3, 4.4)
Wasted in the dry season	12 (8, 17)

1 Values are ORs (95% CIs), based on logistic regression, *n* = 2902 (a subset of participants who visited the clinic at least once during each of their first 3 seasons).

### Early growth trajectories and subsequent risks of stunting

Multilevel models were used to describe longitudinal trajectories of WLZ according to whether the children were stunted or not between the ages of 20 and 24 mo, for the sexes separately. These models were used to predict the growth trajectories for children born on 1 July (start of the wet season), 30 October (end of the wet season), and 1 March (middle of the dry season), which were then plotted to illustrate the relationships between seasonality on growth.

**FIGURE 3:**
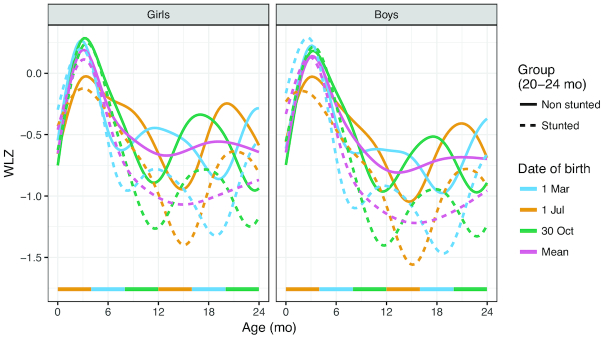
Predicted WLZ trajectories, grouped by stunting status at 20–24 mo, based on mixed-effects growth models, *n* = 5160 participants, 64,342 visits. The mean trajectory (purple) is plotted together with predicted WLZ trajectories for children born at different times of the year, separately among those children who were or were not stunted at 20–24 mo of age. The horizontal colored bars represent ages that coincide with the wet season (July–October), for children born at different times of year. DoB, date of birth; WLZ, weight-for-length *z* score.

**Figure 3** shows the mean WLZ (as purple lines) for children grouped by whether they were stunted between the ages of 20 and 24 mo (dashed line), or not (solid line). Also shown in this figure are the 3 predicted WLZ trajectories for children born at different times of the year, separately for those children who were or were not stunted at 20–24 mo of age. This illustrates the seasonal pattern of weight gain and loss in this population. In both groups (stunted and not stunted), WLZ increases towards the WHO standard during the first 3 mo (as reflected in the decline in wasting prevalence in Table 1 and Figure 2). A distinct separation in WLZ between children stunted and those not stunted at 20–24 mo of age is observed at 5–10 mo of age, although these 2 groups show the same seasonal fluctuations. The predicted WLZ trajectories illustrate how season of birth influences the pattern of subsequent growth in this population. In particular, the downward seasonal effect for subjects born at the start of the wet season (orange lines) partly counteracts the early catch-up of WLZ that was observed among participants born at other times of the year. Among participants born at the start of the wet season, particularly boys, there also appears to be an earlier divergence between the stunted/nonstunted groups.

### Individual weight-for-length trajectories as predictors of becoming stunted

**Table 3** shows ORs from logistic regression models with being stunted at 20–24 mo of age as the outcome. The predictors were sex (boy = 1, girl = 0); any period of being stunted or wasted before the age of 20 mo as binary predictors; and the individual-level random effects estimates taken from multilevel models of the WLZ trajectories (intercept, slope, and CCV). The random effects represent sources of between-individual variation in the trajectories of WLZ.

**TABLE 3:** Odds ratios of stunting at 20–24 mo, based on features of growth before 20 mo^1^

	Model I	Model II	Model III
Predictors			
Baseline odds	0.076 (0.065, 0.089)	0.070 (0.059, 0.082)	0.066 (0.055, 0.079)
Sex (boy)	0.95 (0.82, 1.10)	0.93 (0.80, 1.08)	0.93 (0.80, 1.08)
Ever stunted	15 (13, 18)	14 (12, 17)	13 (11, 15)
Ever wasted		1.4 (1.2, 1.7)	1.3 (1.0, 1.6)
Intercept^2^			0.89 (0.79, 1.00)
Slope^2^			0.00095 (0.00013, 0.00707)
CCV^2^			2.4 (1.2, 4.5)
Diagnostics			
Error rate, %	24	24	24
False-negative rate, %	15	15	18
False-positive rate, %	27	27	26

1 Values are ORs (95% CIs) or percentages, as indicated. ORs (95% CIs) based on logistic regression, *n* = 5160. CCV, coefficient of cyclic variation; WLZ, weight-for-length *z* score.

2 Random effects estimates extracted from WLZ trajectories. The random effects represent individual variation around the mean trajectory of WLZ (with mean zero), e.g., the CCV of the random effects for the Fourier terms quantifies the magnitude of the seasonal variation in WLZ experienced by an individual above/below the seasonal variation described by the average trajectory.

Model I indicates that previous stunting and the sex of the child correctly predicted a child being stunted at 20–24 mo of age in 76% of the sample. More importantly, the false-negative rate was 15%. This is an important measure of model performance, because it represents the proportion of stunted individuals who were not correctly identified. Adding a prior episode of being wasted (model II) did not improve the predictions as individuals identified as at risk of stunting were already identified with the use of model I. However, the ORs in Table 3 show that subjects who had been wasted before the age of 20 mo had 1.4 times greater odds of being stunted at 20–24 mo than those who had not, even after accounting for being stunted at <20 mo of age. Adding the random effects from the growth trajectories (model III) did not change the overall error rate, but as the false-negative rate was 18% it was a little more likely not to identify subjects as nonstunted who do become stunted. A similar analysis was attempted based on a previous period of having a small MUAC instead of a period of being wasted, and applying random effects taken from MUAC trajectories instead of WLZ trajectories. The results of this analysis did not differ meaningfully from the results for WLZ and so are not reported.

### Longitudinal prediction of becoming stunted through the use of time-lagged wasting

**FIGURE 4:**
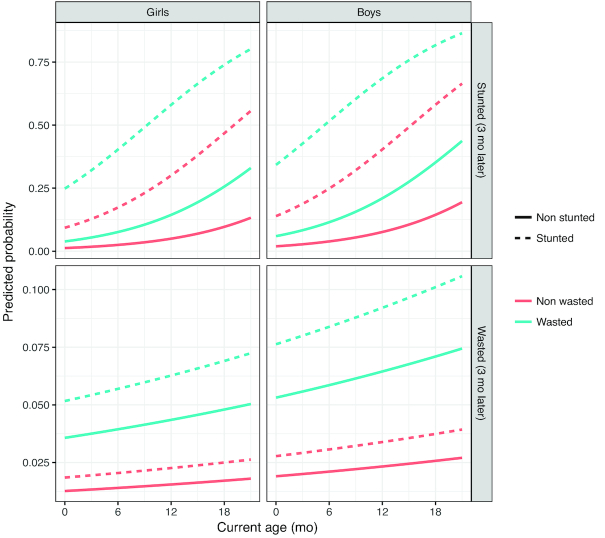
Predicted probabilities of stunting and wasting, based on the models reported in Table 3, *n* = 3867 participants, 28,403 visits. Being stunted increases the probability of being wasted and/or remaining stunted 3 mo later (dashed lines compared with solid lines). Being wasted also increases the probability of being stunted and/or remaining wasted 3 mo later (blue lines compared with red lines). For example, a boy who is both stunted and wasted at 12 mo has a 69% chance of still being stunted and a 9.2% chance of still being wasted at 15 mo, whereas a girl who is neither stunted nor wasted at 18 mo has a 9.7% chance of becoming stunted and a 1.7% chance of becoming wasted by age 21 mo.

Using time-lagged observations, being wasted increases the odds of becoming stunted (3 mo later) by a factor of 3.2, irrespective of whether subjects were already stunted (**Table 4**). Thus, being wasted is predictive of becoming stunted later, even after accounting for current level of stunting. Furthermore, the odds of being stunted were 1.6 times greater among boys than among girls, and each additional month of age increased the odds of being stunted by a factor of 1.1, as the risk increases with age. Of interest, the reverse also holds: children currently stunted are 1.5 times more likely to be wasted 3 mo later, even after accounting for currently wasting status (Table 4). **Figure 4** shows the predicted probabilities of being stunted or wasted after 3 mo, for 4 groups based on stunting or wasting status.

**TABLE 4:** Longitudinal prediction of stunting and wasting based on time-lagged wasting and stunting^1^

	Stunting	Wasting
Baseline odds	0.012 (0.010, 0.016)	0.013 (0.010, 0.016)
Sex (boy)	1.6 (1.3, 1.9)	1.5 (1.2, 1.8)
Age (lagged)	1.1 (1.1, 1.1)	1.0 (1.0, 1.0)
Stunting (lagged)	8.2 (7.2, 9.4)	1.5 (1.3, 1.7)
Wasting (lagged)	3.2 (2.7, 3.9)	2.9 (2.5, 3.4)

1 Values are ORs (95% CIs), based on multilevel logistic regression, *n* = 3867 participants, 28,403 visits. Wasting is predictive of stunting 3 mo later, even after accounting for current stunting. Stunting is also predictive of wasting 3 mo later.

## Discussion

Preventing malnutrition in children aged <5 y is a global priority. The highest prevalence of stunted and wasted children is in south Asia; however, this region has also seen the greatest decline in prevalence. The situation in sub-Saharan Africa is less positive: the prevalence of stunted and wasted children remains high and shows lower rates of decline (23). Globally, no nation is on course to meet all 5 of the 6 global maternal and child nutrition targets, which include the World Health Assembly (WHA) targets to reduce the number of stunted children by 40% by 2025 or wasting to <5% (24). Wasting and stunting are usually separated in terms of policy, guidance, programming, and financing (5). This is largely because wasting is viewed as an acute transient condition amenable to being treated and reversed with the right nutrition and medical interventions. Stunting, however, is viewed as chronic in nature, largely irreversible after 2 y of age, and is associated with long-term consequences on adult human capital (25), and so needs to be prevented. Understanding the relationships between these 2 outcomes of undernutrition may help to increase the effectiveness of interventions towards both outcomes and help achieve the WHA targets.

In this population of rural Gambian infants and young children, the prevalence of wasting or stunting was high, and showed age-related trends typical of other low- and middle-income settings (26, 27). Gains in WLZ were observed in the first few months of postnatal life, followed by a period of rapid decline. The prevalence of children who were wasted peaked at ∼12 mo of age in both boys and girls and prevalence rates of being stunted increased to a peak at 20–24 mo of age. The prevalence of children who were both concurrently wasted and stunted peaked at ∼9% for boys and ∼5% for girls at ∼12 mo of age and declined thereafter. Estimates of the prevalence and burden of children concurrently wasted and stunted have only recently been made and report a prevalence from 84 country datasets ranging from 0% to 8% (28).

The decline in the proportion of concurrence from ∼12 mo in girls and from ∼16 mo in boys may be the result of stunted subjects gaining weight or failing to grow in length while remaining the same weight, so they were no longer classified as wasted; this situation, therefore, does not result in an overall decline in the prevalence of stunting. From a physiologic point of view, slowing of linear growth may represent an “internal” adjustment to resolve wasting by diverting resources to tissue accretion rather than overall size increase. In this sense, concurrence may be viewed as transient if stunting leads to an apparent “recovery” of weight-for-length, but it may also represent a useful marker of such a slowing of linear growth.

Although wasting is commonly viewed as being transient and treatable, our results indicate that its effects are longer term through being “reabsorbed” into linear growth slowing. By grouping children according to whether they are stunted or not stunted at ∼2 y of age, we have shown that their mean WLZ appears to separate when the children are between 5 and 10 mo of age. As the general trend in children in this community is for rapid growth faltering during this period of life (15, 26), this observation simply highlights a more rapid decline in WLZ for children who become stunted in later childhood. This finding is, perhaps, intuitive, but highlights the particular opportunity of intervention early in life, to avoid a greater decline in WLZ between 5 and 10 mo to reduce the risk of later stunting.

An unexpected finding that emerged from this analysis, however, was that, although WLZ catches up to the WHO median during the first 3 mo (as shown in Figure 3), this catch-up was smaller in subjects born at the start of the wet or “hungry” (lean) season. This seasonally associated reduction in weight gain was more marked among boys than girls, who experienced less catch-up as well as an earlier divergence when separated into groups who are eventually stunted and not stunted. In this population, exclusive breastfeeding to 4–6 mo of infant age is almost universal, with continued partial breastfeeding thereafter (29). A seasonal influence on infant feeding practices in this context has not been observed, and although this remains possible, this finding may also reflect a biological response to the differing seasonal environment. We have previously observed a seasonal influence on human milk oligosaccharides, with the suggestion that these seasonal differences correlate with infant development of the gut microbiome and infant growth (30). It may also be explained by a higher incidence of infectious diseases during the wet season.

A common theme throughout the analyses presented here is the greater susceptibility among boys than girls to becoming wasted or stunted and becoming both concurrently wasted and stunted. This observation is consistent with data from other low-income populations where boys typically exhibit higher rates of undernutrition than girls. In a meta-analysis of 16 Demographic and Health Surveys from sub-Saharan Africa, Wamani et al. (31) demonstrated that boys aged <5 y are more likely to become stunted than girls (age- and survey-adjusted OR: 1.18; 95% CI: 1.14, 1.22), with evidence to suggest that this sex difference was more pronounced in the lowest socioeconomic groups. A recent analysis of data from 84 countries also concluded overall that boys were significantly more likely than girls to be both wasted and stunted (28). It is possible, although unlikely given the widespread nature of this observation, that there may be issues with the construction of the growth curves leading to a sex bias. It is more likely, however, that there are social or physiologic factors that make boys more susceptible to undernutrition than girls. For example, optimization of maternal reproductive success can select for male vulnerability in poor environments, and this evolutionary trade-off may manifest itself as greater susceptibility to stresses, including undernutrition (32). This scenario applies to fetal life as well as infancy (33), but intriguingly, India shows a contrasting pattern, which may be due to cultural factors favoring sons (34). Although it would be programmatically difficult to target boys, an understanding of whether their vulnerability is an artifact of the standards or whether there are real etiological drivers of this increased susceptibility can help inform more targeted interventions.

Based on the use of an age-dependent longitudinal model to predict stunting, we observed that being wasted increases the odds of becoming stunted by 3.2 times, irrespective of whether the child was already stunted 3 mo earlier. Of particular interest, and not reported elsewhere in the literature, the reverse was also observed, with a stunted child also more likely than a nonstunted child to be wasted 3 mo later although with lower odds. This tells us that the relation between these 2 outcomes of malnutrition is not a simple or linear one. Here, we have not investigated potential common drivers of wasting and stunting, although previous reviews conclude there are many overlaps indicating extrinsic factors are also at play (7). Further understanding this relationship should be a priority for future research.

The tendency to pay attention to wasting in humanitarian response focused on its treatment and to stunting in development contexts because it needs to be prevented, rather than to both manifestations, highlights a need to reconceptualize how we deal with wasting and stunting. We need to forge a more joined-up approach to treatment and prevention in all contexts in which these manifestations of undernutrition are prevalent and across the academic, policy, and practice communities.

In conclusion, this analysis, which used longitudinal data from children in rural Gambia spanning 4 decades, has highlighted several key issues of relevance to our understanding of the relationship between wasting and stunting in early childhood. First, in this highly seasonal, rural environment with high rates of exclusive breastfeeding, we have identified a seasonally driven risk among young infants to poor growth, indicating the need to provide more targeted support to breastfeeding mothers and increased attention to infant feeding during periods of seasonal stress. Second, we have demonstrated that being wasted leads to increased risk of subsequent stunting. Third, we have demonstrated that children who are wasted in 1 wet season are more likely to be wasted in a subsequent wet season even after recovery, suggesting a continued vulnerability across seasons requiring further understanding of the physiologic mechanisms and environmental factors. Fourth, and consistent with much of the global literature, boys are more likely to be wasted or stunted, both of which convey added risk of mortality. In sub-Saharan African, there is a substantially higher <5-y mortality rate among boys than among girls (107 compared with 69 per 1000 live births) (35), highlighting the need to understand this sex difference in vulnerability so that the policy and practice community can take this into account. In addition, we have demonstrated that concurrence is more prevalent in boys and in the younger child, which carries an even higher risk of death, a finding that is similar to reports in other studies cited in this paper.

Our results indicate that where there are levels of wasting and stunting of public health significance in a given context, there are compelling reasons for both treatment and prevention interventions to consider them jointly and with awareness of the relation between them. The separation of the wasted infant/child and the stunted infant/child in terms of policies, programs, and research risks opportunities being missed to detect and intervene to prevent both forms of undernutrition in this highly vulnerable population group. The attainment of WHA and other global targets remains a very strong global and country-level intent, but these targets will not be achieved as long as our approaches to infant and child undernutrition remain siloed.

## Notes

The analysis presented in this paper was funded by the United States Agency for International Development (USAID), grant ENN AID-OFDA-G-15-00190, and Irish Aid. The contents of this paper are the responsibility of the authors and do not necessarily reflect the views of USAID, NIH, or the US Government, nor those of Irish Aid or the Irish Government. The funders had no role in study design, data collection, analysis, decision to publish, or preparation of the manuscript. The MRC International Nutrition Group is supported by the UK Medical Research Council (grant MC-A760-5QX00) and the UK Department for International Development under the MRC-DFID Concordat agreement.

Abbreviations used: CCV, coefficient of cyclic variation; LAZ, length-for-age *z* score; MRC, Medical Research Council; MUAC, mid-upper-arm circumference; WLZ, weight-for-length *z* score; WHA, World Health Assembly
